# Insight into Cisplatin-Resistance Signaling of W1 Ovarian Cancer Cells Emerges mTOR and HSP27 as Targets for Sensitization Strategies

**DOI:** 10.3390/ijms21239240

**Published:** 2020-12-03

**Authors:** Kathleen Wantoch von Rekowski, Philipp König, Svenja Henze, Martin Schlesinger, Piotr Zawierucha, Radosław Januchowski, Gerd Bendas

**Affiliations:** 1 Department of Pharmacy, University of Bonn, 53113 Bonn, Germany; kathleen.wantoch@uni-bonn.de (K.W.v.R.); philippkoenig@uni-bonn.de (P.K.); svenja.henze@uni-bonn.de (S.H.); martin.schlesinger@uni-bonn.de (M.S.); 2 Department of RNA Metabolism, Institute of Bioorganic Chemistry Polish Academy of Sciences, 61-704 Poznań, Poland; pzawierucha@ibch.poznan.pl; 3 Institute of Health Sciences, Collegium Medicum, University of Zielona Gora, Zyty 28 St., 65-046 Zielona Góra, Poland; rjanuchowski@ump.edu.pl

**Keywords:** CAM-DR, cisplatin, collagen, ovarian cancer, chemoresistance, HSP27

## Abstract

The microenvironment possesses a strong impact on the tumor chemoresistance when cells bind to components of the extracellular matrix. Here we elucidate the signaling pathways of cisplatin resistance in W1 ovarian cancer cells binding to collagen type 1 (COL1) and signaling interference with constitutive cisplatin resistance in W1CR cells to discover the targets for sensitization. Proteome kinase arrays and Western blots were used to identify the signaling components, their impact on cisplatin resistance was evaluated by inhibitory or knockdown approaches. W1 cell binding to COL1 upregulates integrin-associated signals via FAK/PRAS40/mTOR, confirmed by β1-integrin (ITGB1) knockdown. mTOR appears as key for resistance, its blockade reversed COL1 effects on W1 cell resistance completely. W1CR cells compensate ITGB1-knockdown by upregulation of discoidin domain receptor 1 (DDR1) as alternative COL1 sensor. COL1 binding via DDR1 activates the MAPK pathway, of which JNK1/2 appears critical for COL1-mediated resistance. JNK1/2 inhibition inverts COL1 effects in W1CR cells, whereas intrinsic cisplatin resistance remained unaffected. Remarkably, knockdown of HSP27, another downstream MAPK pathway component overcomes intrinsic resistance completely sensitizing W1CR cells to the level of W1 cells for cisplatin cytotoxicity. Our data confirm the independent regulation of matrix-induced and intrinsic chemoresistance in W1 ovarian cancer cells and offer novel targets for sensitization.

## 1. Introduction

Ovarian cancer is the deadliest malignancy among gynecological tumors and ranks 7th among the most frequently diagnosed carcinomas in women [1,2]. The high mortality is on the one hand based on the late diagnosis of this malignancy, since symptoms often appear after the tumor has formed metastases. The standard treatment of ovarian cancer is a platinum-based or paclitaxel chemotherapy following a maximum cytoreductive surgical neoadjuvant therapy [3,4]. On the other hand, resistance formation against these cytotoxic drugs appears another obstacle in treatment. Although the majority of patients initially respond well to these antineoplastic drugs, ovarian cancer has a high recurrence rate, which often goes along with resistance formation. However, a change in drug regime, including liposomal doxorubicin, topotecan, or gemcitabine cannot overcome resistance phenomena. This impressively illustrates the versatility of resistance mechanisms, ranging from mutational hyper-activation of proliferation signaling to changes in drug trafficking and metabolism to circumvent apoptosis. Accordingly, the relative 5-year survival rate of just 41% is comparably low [5].

It is known that the microenvironment of tumors affects the cytotoxic effectiveness of drugs. Adhesion receptors on cancer cells mediate a resistance by binding to components of the extracellular matrix (ECM) and subsequently activate downstream signaling pathways. This process, also referred to as cell adhesion-mediated drug resistance (CAM-DR) has been confirmed in its relevance for different tumor cell lines representing models for various tumor entities [6,7,8]. We have shown before that CAM-DR occurs in the ovarian cancer cell line W1 affecting their cisplatin sensitivity and interestingly adds to the already existing cisplatin resistance in the W1CR variant of these cells when binding to COL1 [9]. Nevertheless, molecular mechanisms of CAM-DR in W1 cells and their interplay with cisplatin resistance in W1CR cells remain ambiguous. Considering the key role of CAM-DR as an initial form of resistance formation, an insight into the underlying molecular pathways could provide novel targets for early pharmacological interference with resistance formation [10,11].

CAM-DR has been associated with integrins, transmembrane adhesion receptors of heterodimeric α- and ß-subunits [10,11]. Integrin binding to a substrate activates different signaling pathways within the cell via adapter proteins and protein kinases [12]. A change or disruption in the molecular process of focal adhesion assembly and thus, also of cell-matrix adhesion can lead to a malignant transformation of cells [13]. Integrin signaling is mediated via different pathways, often initiated by the focal adhesion kinase (FAK) associated with integrin-linked kinase (ILK), phosphoinositide 3-kinase (PI3K), and protein kinase B (AKT). The activity of PI3K as a key molecule at the interface of multiple cell signaling pathways [14,15] is regulated by the phosphatase and tensin homolog (PTEN), which is considered as the tumor suppressor mutationally inactivated in many tumors [16,17]. The mechanistic target of rapamycin (mTOR) represents another downstream key component of PI3K/AKT, which acts as a growth regulator for protein translation with consequences for cell proliferation [18,19]. The connection of mTOR and PI3K/AKT is mediated by prolin-rich AKT substrate of 40 kDa (PRAS40), an AKT substrate, and simultaneously component of mTORC1 [20]. Overexpression or hyper-phosphorylation of PRAS40 has been reported in a variety of tumors [21,22,23].

Although β1-integrins (ITGB1) appear the dominant cellular collagen receptors, discoidin domain receptors 1 and 2 (DDR1, DDR2) act independently of integrins as receptor tyrosine kinases (RTK) [24] in binding predominantly COL1, 3, and 4. DDRs have become increasingly relevant as potential targets in cancer [25]. DDR1, expressed in many epithelial and carcinoma cells, has been linked to the invasion of cancer cells in 3D collagen, primarily by promoting the production of matrix metalloproteases (MMPs) [26,27]. Notably, signaling processes initiated by DDR1 are similar to those of integrins and have recently been associated with CAM-DR in breast cancer cells [28].

Integrins and DDR1 share, besides PI3K/AKT and mTOR signaling, the MAPK pathway to control cell growth and survival. The MAPK pathway can act via three functional arms. These include extracellular signal-regulated kinase (ERK), c-Jun-N-terminal kinase (JNK), and p38 mitogen-activated protein kinase (p38-MAPK) [29,30]. In cancer cells, JNK maintains the expression of pro-survival proteins, e.g., survivin [31], thereby increasing the resistance to cisplatin treatment [32,33]. JNK responds, among other triggers, to un- or misfolded protein stress, which is a frequent issue in cancer cells [34,35]. Protein folding is usually mediated by chaperone molecules like heat shock proteins (HSPs) that are involved in the correct folding and maturation of proteins. HSPs were classified according to their molecular mass, such as HSP27 [36,37]. Cellular stress induces a rapid increase in HSP27 phosphorylation within minutes and increased the expression after several hours. HSP27 interferes at the outer mitochondrial membrane with the activation of the cytochrome complex and thus inhibits the activation of procaspase-9 [38]. Overexpression of HSP27 is closely related to metastasis, invasiveness, and poor prognosis in various cancers [39,40]. Increased expression of HSP27 is also found to be associated with resistance to chemotherapy [41].

In the present study, we aim to elucidate the underlying mechanisms of COL1-induced loss in cisplatin sensitivity in W1 ovarian cancer cells, and how this is functionally combined with existing cisplatin resistance in the W1CR subline. Using an ITGB1 knockdown variant we show that COL1 binding of W1 cells strictly depends on this integrin fostering mTOR activity as key for loss of cisplatin sensitivity. The blockade of mTOR sensitizes W1 cells reversing the COL1 effect. In contrast, resistant W1CR cells follow, despite comparable CAM-DR effects upon COL1 binding, totally different routes demonstrating a highly dynamic interplay of ITGB1 and DDR1. Following the DDR1/ITGB1-induced MAPK pathway, JNK1/2 appears crucial for CAM-DR, whereas HSP27 is the key component for permanent resistance. Our study provides an insight into the complex resistance signaling and novel targets for sensitization.

## 2. Results

### 2.1. Impact of COL1 Binding on Cellular Signaling Pathways in Cisplatin-Treated W1 and W1CR Ovarian Cancer Cells

W1 ovarian cancer cell line and its cisplatin-resistant subtype W1CR, which is indicated by roughly a seven-fold higher EC_50_ value against cisplatin cytotoxicity, further increase resistance against cisplatin more than two-fold when they were cultivated on COL1 [9]. Although this CAM-DR phenomenon appears similar in both cell lines, the underlying molecular mechanisms are unknown and seemingly different, considering a clear dependency on ITGB1 in W1 cells, but an obvious independence on ITGB1 in W1CR cells [9]. In this recent study we also analyzed the genome data of W1 and W1CR cells treated with COL1 and/or cisplatin at the mRNA level [9].

Here, we initially re-analyzed this data set to search for remarkable deregulation of potential signaling components. However, no clear deregulation of kinases or other signaling components were evident in the COL1-treated cells to favor an obvious signaling pathway already at the mRNA level [9]. Nevertheless, we refer to this data set for selected components later on in our investigations.

**Figure 1:**
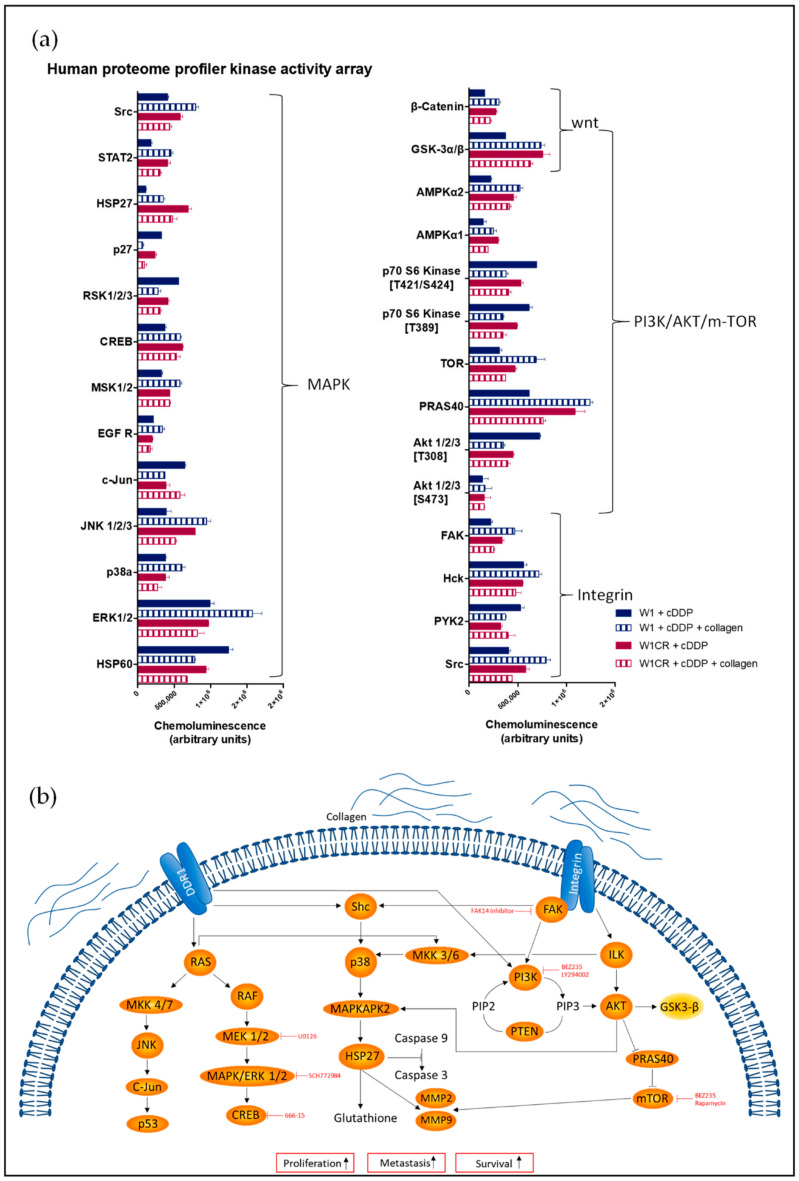
(**a**) Data set of a human proteome profiler kinase activity array displaying the phosphorylated/activated form of the components in cisplatin (cDDP)-treated W1 and W1CR cells with and without collagen (*n* = 1). (**b**) Overview of the investigated signaling pathway and inhibitory approaches.

To provide a closer insight into the signaling pathways associated with COL1-induced resistance at the protein level, we performed a proteome profiler array focusing on kinome analysis in cisplatin-treated W1 and W1CR cells in the absence or presence of COL1. Data clearly indicated that COL1 binding upregulated the majority of signaling components in W1 cells. In contrast, W1CR signaling was generally more downregulated upon COL1 binding (Figure 1a). Nevertheless, W1CR cells display an intrinsically more intensive signaling under cisplatin treatment than W1 cells (solid columns), which exceeds in case of GSK3α/β and HSP27 also the level of COL1-treated W1 cells. However, a clear matrix binding–resistance axis is not obvious at a first view.

Having an integrin background in mind, the data did not directly highlight a signaling pathway traditionally associated with integrins (Figure 1b). However, key components of integrin signaling were analyzed in detail concerning their involvement in resistance formation and their value as targets for sensitization strategies.

### 2.2. The Role of the FAK/PI3K/AKT Pathway for COL1-Induced Cisplatin Resistance Formation in W1 and W1CR Cells

**Figure 2:**
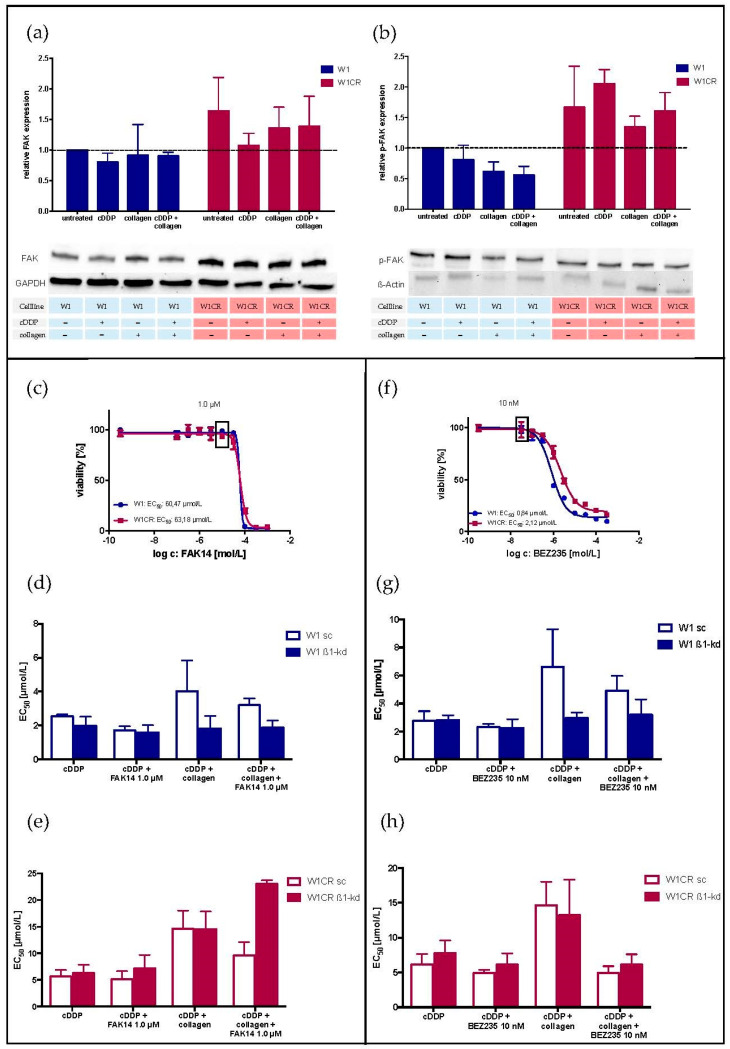
The impact of FAK and PI3K on resistance formation in W1 and W1CR cells as a potential target for sensitization: the expression of FAK (**a**) and p-FAK (**b**) in W1 and W1CR cells upon the indicated treatment regime was detected by Western blot. (**c**) Cytotoxicity of FAK14 in W1 and W1CR cells to select a non-toxic concentration of 1 µM to be used for a co-treatment with cisplatin. (**d,e**) Blocking FAK (1.0 µM FAK14) and the impact on cisplatin toxicity in W1 scramble or W1 ITGB1 knockdown cells (**d**) and W1CR scramble and W1CR ITGB1 knockdown cells (**e**). (**f**) Cytotoxicity of the PI3K inhibitor BEZ235 in W1 and W1CR cells to determine a non-toxic concentration of 10 nM for combination with cisplatin treatment in these cells. (**g,h**) Cisplatin cytotoxicity upon PI3K inhibition (10 nM BEZ235) in W1 scramble and W1 ITGB1 knockdown cells (**g**) and W1CR scramble and W1CR ITGB1 knockdown cells (**h**). Data are means of at least *n* = 3 (±SEM).

The PI3K/AKT activation pathway, which is often initiated by an integrin-mediated clustering of FAK, has been associated with increased cell proliferation and survival upon cytotoxic stress [18]. To evaluate the impact of this pathway, we initially focused on FAK. It became clear that the level of FAK is even higher in W1CR cells (Figure 2a). Although activated p-FAK was also higher (Figure 2b) in W1CR cells, activation of FAK in dependency of cisplatin treatment or COL1 binding was not evident in both cell lines. Applying a FAK-inhibitor (FAK 14) at non-toxic concentrations of 1 µM (Figure 2c) had hardly any effect on reversing the COL1-induced cisplatin resistance in W1, and a more pronounced, but yet not significant sensitizing effect in W1CR cells (Figure 2d,e). Notably, the knockdown of ITGB1 below 10% in W1 cells (Figure S1) bypassed the COL1 effect on resistance and any effects of FAK14 completely, indicating a clear COL1/ITGB1/FAK axis. More interestingly, when knocking down ITGB1 in W1CR cells to a residual level below 10% (Figure S1), the kd variant maintained COL1 resistance and lost sensitization by FAK14. One might assume an alternative COL1 sensor beside ITGB1 in these cells.

Concerning PI3K, both cell lines express comparable levels of PI3K and pPI3K, which were not upregulated by cell treatment with cisplatin or even COL1 binding (Figure S2a,b). Notably, COL1 binding decreased the pPI3K level in W1 cells more evidently than in W1CR cells. To further focus on the functional role of PI3K, we applied the dual PI3K/mTOR inhibitor dactolisib (BEZ235) at a non-toxic concentration of 10 nM (Figure 2f). The COL1 effect on reduced cisplatin sensitivity was marginally reversed by BEZ235 in W1 cells, while knockdown of ITGB1 circumvented any COL1 effects but also BEZ235 activities (Figure 2g), similar to the finding on FAK in these cells. In contrast, BEZ235 reversed the COL1 effect in W1CR cells and the ITGB1 kd variant completely (Figure 2h), supporting the assumption on the independence on ITGB1, but however involvement of PI3K. LY294002, another PI3K inhibitor displayed comparable data referring to a slight sensitization of the COL1 effect in W1 and W1CR cells to cisplatin (Figure S2c).

**Figure 3:**
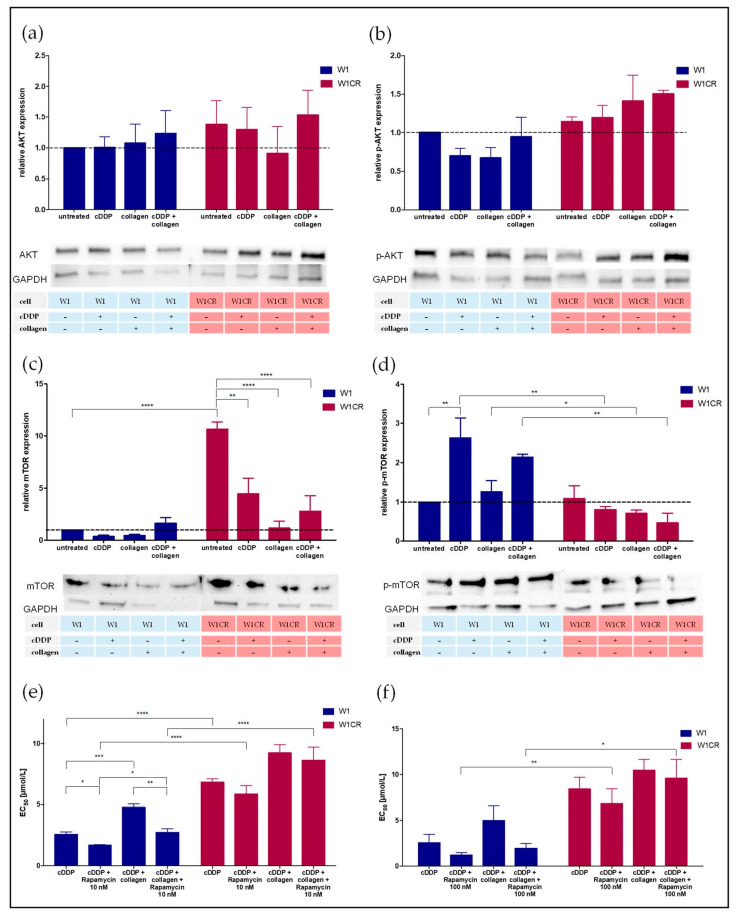
An insight into the AKT/mTOR signaling axis for W1 and W1CR cells. (**a**–**d**) Western blot of W1 and W1CR cells to illustrate the expression of AKT (**a**), p-AKT (**b**), mTOR (**c**), and mTOR in its phosphorylated form (**d**). Inhibition of mTOR in W1 and W1CR cells by rapamycin, which specifically blocks mTORC1 in a concentration of 10 nM (**e**) and 100 nM (**f**) and its impact on cisplatin sensitivity. Data are means of at least *n* = 3 (±SEM), asterisks indicate statistical significance: * *p* < 0.05; ** *p* < 0.01; *** *p* < 0.001; **** *p* < 0.0001.

The higher impact of PI3K on resistance signaling in W1CR cells was also reflected by higher protein levels of AKT and p-AKT as downstream components, compared to W1 cells (Figure 3a,b). Probably, the PI3K/AKT axis in W1 cells was antagonized by higher expression of the phosphatase PTEN (Figure S2d,e), which counteracts in PI3K-mediated PIP3 formation by cleaving a phosphate from PIP3 and thus functionally antagonizing AKT activation. Remarkably, the highest levels of PTEN in W1 cells were detected upon cisplatin and COL1 treatment.

Together, W1CR cells upregulate the PI3K/AKT pathway with a certain impact on COL1-induced CAM-DR, but obviously independent on ITGB1. In contrast, W1 cells follow an ITGB1 signaling via this pathway, but the resistance formation is apparently mediated via another, probably downstream component.

### 2.3. Insight into AKT/mTOR Axis Emerges mTOR as a Key Target for W1 Cell Sensitization

To follow AKT further downstream, we evaluated the activation of GSK3α/β as AKT substrates offering a potential link to Wnt signaling, which was associated with increased malignancy or resistance formation in cancer cells [42]. However, analyzing GSK3α and β in the activated form (Figure S3a,b) neither provides a significant impact of the cell treatments nor differences between W1 and W1CR cells. This is in agreement with the former findings on the inability of the Wnt inhibitor FH535 to affect W1CR cisplatin resistance. Thus, the Wnt signaling pathway was excluded as a probable key player in the COL1-mediated resistance formation of these cells.

mTOR, another downstream target of AKT is a key mediator for cell growth and proliferation [18]. The AKT/mTORC1 pathway is regulated by PRAS40, which represents an inhibitory component of the mTORC1 complex. As a substrate of AKT, and partly of mTORC1 itself, phosphorylation of PRAS40 alleviates the mTOR inhibition and consequently activates the AKT/mTORC1 pathway. Interestingly, W1 cells displayed a strong upregulation of activated/phosphorylated PRAS upon COL1 binding, whereas W1CR cells attenuated PRAS activation thereof (Figure 1a). For further insight into mTOR activities, we analyzed mTOR in both cell lines (Figure 3c,d). Although the level of mTOR was much higher in W1CR cells, activation of mTOR by phosphorylation was evidently higher in W1 cells. Notably, cisplatin treatment further increased the p-mTOR levels in W1 cells, while cisplatin and COL1 further decreased p-mTOR in W1CR cells.

To functionally reflect the indicated higher activity of mTOR in W1 cells, we performed mTOR inhibition applying rapamycin. Impressively, rapamycin in a low and a higher concentration (10 nM or 100 nM, respectively) was able to totally reverse the COL1-mediated resistance in W1 cells (Figure 3e,f), while W1CR cells were not affected by rapamycin upon COL1 binding.

These findings highlight mTOR as a critical target for the sensitization of CAM-DR induced by COL1 in W1 cells. In contrast, COL1 effects in W1CR cells seemingly follow another resistance route.

### 2.4. DDR1 Is a COL1 Sensor in W1CR Cells

Our data show that W1CR cells keep their COL1-induced cisplatin resistance even under an ITGB1 knockdown, indicating the activity of other collagen receptors. Only a few COL1 receptors are described in literature besides ITGB1. Among them, DDR1 appears as the most probable candidate, since the aberrant expression of DDR1 was associated with increased tumorigenicity in different cancer entities [25,43]. To elucidate whether DDR1 is a relevant receptor in W1CR cells, we analyzed the expression levels of DDR1 and p-DDR1 by Western blot and compared them with those in W1 cells. In general, the level of DDR1 is roughly three-fold higher in W1CR vs. W1 cells.

**Figure 4:**
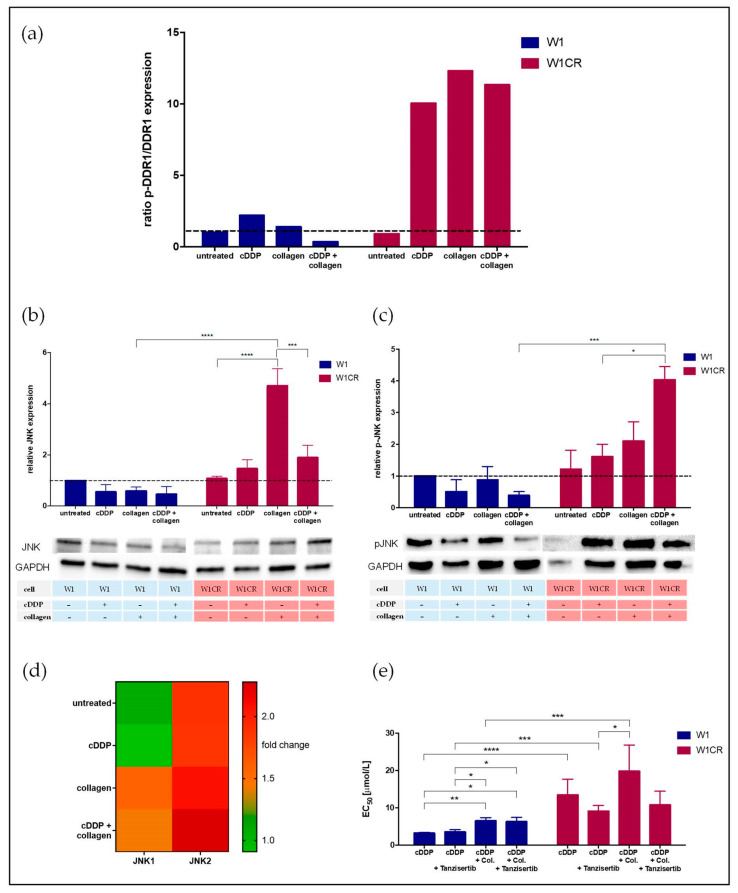
The impact of DDR1 on CAM-DR in W1 and W1CR cells. (**a**) Western blot data of DDR1 activation under the influence of cisplatin and/or collagen indicated by the protein expression ratio of p-DDR1/DDR1. JNK protein levels (**b**), and the corresponding activated p-JNK levels of W1 and W1CR cells (**c**). (**d**) Higher JNK expression in COL1-treated W1CR cells is also reflected at the mRNA level, indicated by the two-fold deregulation vs. W1 cells shown as fold change. (**e**) The effect of JNK inhibition by tanzisertib (1 µM) to affect cisplatin-sensitivity in W1 and W1CR cells indicate a key role of JNK for COL1-mediated resistance in W1CR cells, but not W1 cells. Data are means of at least *n* = 3 (±SEM), asterisks indicate statistical significance: * *p* < 0.05; ** *p* < 0.01; *** *p* < 0.001; **** *p* < 0.0001.

Considering the ratio of p-DDR1 and DDR1 expression in both cells as an indicator for DDR1 activation status (Figure 4a), cisplatin treatment and even more COL1 induce a massive activation of DDR1 in W1CR, but not in W1 cells. Obviously, W1CR cells make use of DDR1 as an alternative COL1 sensor for mediating matrix-induced resistance, which thus explains the independence from ITGB1 but higher activity via PI3K/AKT in these cells, since PI3K and AKT represent downstream components of DDR1 (Figure 1b).

### 2.5. JNK Appears as Crucial Downstream Component of COL1/DDR1 Resistance Axis in W1CR Cells

DDR1 mediates its protumorigenic activities via different signaling routes, and besides the “classical” MAPK pathway, the signaling via JNK/c-Jun was considered predominantly, since this is known to be involved in matrix-related signaling cascades [43]. JNK has been associated with increased tumor development or, on the contrary, as a promotor of cell stress responses and apoptosis trigger. In light of these findings, we analyzed the protein expression of JNK1/2 in W1 and W1CR cells and the impact of COL1 thereon.

While the basic protein expression levels of JNK1/2 did not differ in untreated W1 and W1CR cells (Figure 4b), COL1 induced a massive upregulation of JNK only in W1CR cells, which was also evident at the mRNA level of these cells (Figure 4d). This was also reflected by higher levels of the activated form of JNK in W1CR cells (Figure 4c). On the contrary, p-JNK was diminished in W1 cells under those treatments. This indicates a key role of JNK in mediating COL1-induced resistance in W1CR cells. To confirm this, we applied tanzisertib (1 µM), which displays inhibitory capacities in the nanomolar range to JNK1/2/3. Notably, while JNK inhibition had no effect on COL1-induced resistance in W1 cells, tanzisertib reversed the COL1 effect in W1CR cells completely (Figure 4e).

Together, these data indicate the critical role of JNK for mediating a COL1-related resistance of W1CR cells and provide a potential target for sensitization and overcoming CAM-DR in these cells. Nevertheless, interfering with JNK activity did not affect the intrinsic resistance of W1CR cells, compared to W1 cells.

### 2.6. MAPK Pathway Did Not Provide Further Targets for Sensitizing Intrinsic Resistance in W1CR Cells

MAPK pathway is regarded as the most prominent signaling pathway triggering protumorigenic activities in cancer cells. The MAPK pathway is altered in approx. 40% of all human cancers, mainly because of mutations in the upstream activator RAS [44]. To elucidate whether the MAPK pathway is also upregulated in terms of the COL1-induced resistance of W1CR cells or upregulated independently of matrix effects as a key for their intrinsic cisplatin resistance, we analyzed the key components MEK and ERK at mRNA and protein expression levels in both cell lines. Furthermore, we applied inhibitory approaches of MEK and ERK and detected consequences for cisplatin sensitivity.

Interestingly, the mRNA level of MEK in untreated W1CR cells was more than three-fold higher compared to W1 cells (Figure S4a). This was reflected at the protein level, considering the activated p-MEK, since the level of p-MEK in untreated W1CR cells exceeded that in W1 cells more than two-fold (Figure S4b). Notably, neither COL1 binding nor combination with cisplatin increased the p-MEK levels indicating an intrinsic high activity in W1CR cells independent of COL1 matrix effects. A non-toxic concentration of the MEK1/2 inhibitor U0126 did not affect W1 and had only a marginal effect on cisplatin sensitivity in W1CR cells (Figure S4c). Analyzing the downstream component ERK, illustrated as a ratio of p-ERK/ERK (Figure S4d), confirmed the higher activity in W1CR cells in an untreated status, which was rather downregulated upon COL1 binding. However, blocking ERK by SCH772984 did not induce sensitization of W1CR cells for cisplatin.

To further follow this pathway, we also focused on CREB as a downstream component of ERK. Although W1 and W1CR cells expressed comparable protein levels of CREB (Figure S5a), W1CR cells strongly exceeded W1 cells in CREB activation by roughly two-fold higher levels of p-CREB (Figure S5b). Notably, as expected from the MEK and ERK data, also p-CREB was not further upregulated by COL1 demonstrating independence of this pathway from COL1. Nevertheless, a blockade of CREB using the small molecule inhibitor 666-15 had no sensitizing effects in W1CR cells (Figure S5c).

These data indicate much higher activity of the MEK/ERK/CREB pathway in W1CR cells, which is obviously independent of COL1 binding but contributes to the intrinsically higher cisplatin resistance. However, the indicated signaling components appear irrelevant for a sensitization.

### 2.7. HSP27 Appears as Central Key for W1CR Resistance

In a further approach to identify the key mechanisms of intrinsic cisplatin resistance of W1CR cells, we refer to the much higher levels of HSP27 in these cells upon cisplatin treatment compared to W1 cells (Figure 1a). It is known that HSP27 as modulator of oxidative cell stress is associated with the level of glutathione. Notably, we have shown that W1CR cells exceed W1 cells by a more than three-fold higher level of glutathione [9]. HSP27 upregulation in different tumor cells has been described as an anti-apoptotic factor for increased proliferation, metastasis, and resistance against cytotoxic stress [45]. Other studies have shown that the MAPK/ERK and the PI3K/AKT signaling pathway regulate the phosphorylation of HSP27 [46,47], which potentially links HSP27 to the context of this study.

**Figure 5:**
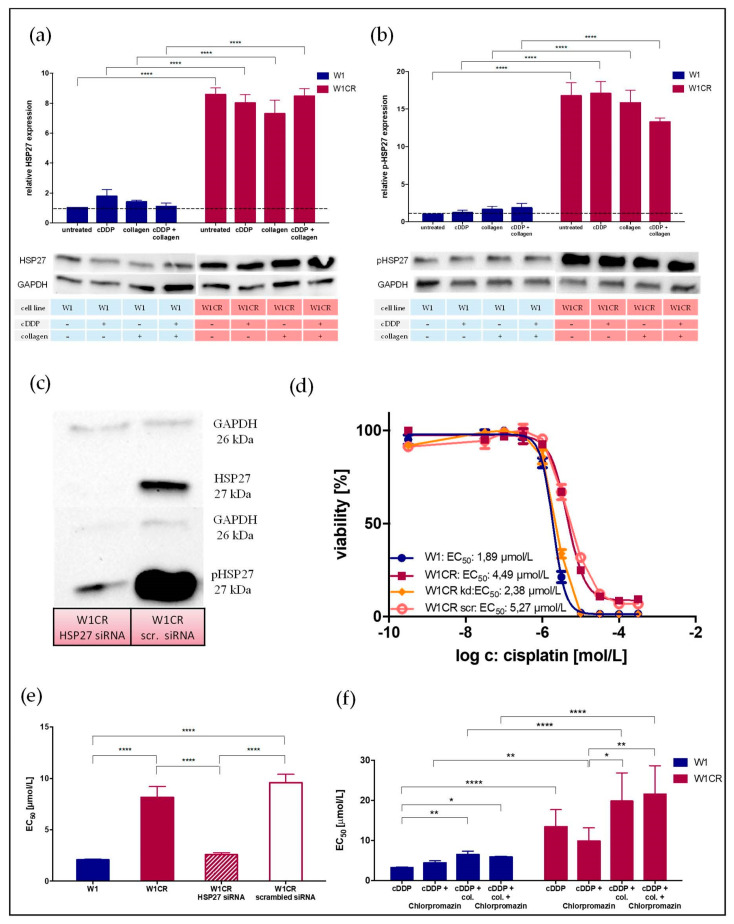
The role of HSP27 in W1CR cell resistance. Detection of protein levels of HSP27 (**a**) and p-HSP27 (**b**) in W1 and W1CR cells upon indicated treatment. (**c**) Knockdown of HSP27 in W1CR cells. Shown is a representative Western blot of HSP27 knockdown in W1CR cells, confirming the almost complete deletion of HSP27 in the knockdown cells compared to scrambled siRNA control. (**d**,**e**) The W1CR HSP27 knockdown cells display a similar sensitivity to cisplatin cytotoxicity as W1 cells, (**d**) shown in a representative MTT experiment and (**e**) statistically evaluated analyzing the EC50 values compared to the scrambled control cells and to W1CR wild type cells. (**f**) Use of chlorpromazine as an inhibitor of HSP27 confirms that W1 cells were not affected, while W1CR cells were sensitized independent of their COL1 effects. Data are means of at least *n* = 3 (±SEM), asterisks indicate statistical significance: * *p* < 0.05; ** *p* < 0.01; **** *p* < 0.0001.

A comparison of HSP27 and activated p-HSP27 protein levels (Figure 5a,b) in both cell lines illustrated a much higher expression of HSP27 in W1CR cells. The level of p-HSP27 in W1CR cells exceeded that in W1 cells by roughly 15-fold. Thus, HSP27 appeared as a potential key for the W1CR cisplatin resistance, independent from COL1-induced effects. To focus on the role of HSP27 at a functional level, we performed a siRNA knockdown approach of HSP27 in W1CR cells. As indicated (Figure 5c), compared to the scrambled control, the W1CR*_HSP27siRNA_* cell variant was nearly deficient in HSP27 and accordingly in p-HSP27 expression. To investigate the effect of HSP27 knockdown in W1CR cell, cisplatin cytotoxicity was analyzed. While the scrambled control cells displayed nearly identical EC_50_ values compared to the W1CR wild-type cells, the W1CR*_HSP27siRNA_* cells behaved comparably to W1 cells with respect to cisplatin sensitivity (Figure 5d,e). Notably, a total loss of resistance in W1CR*_HSP27siRNA_* cells was detectable.

To further confirm the key role of HSP27 in cisplatin resistance we aimed to block it. The antipsychotic drug chlorpromazine has recently been described as a non-specific inhibitor of HSP27 [48]. Therefore, we applied chlorpromazine at a 10 µM concentration and detected the impact on cisplatin resistance of W1CR cells (Figure 5f). Interestingly, chlorpromazine had no effect on W1 cell sensitivity to cisplatin and slightly increased EC_50_ values leaving COL1 effects unaffected. In striking contrast, chlorpromazine attenuated W1CR cell resistance evidently but did not affect the COL1 impact on resistance. This sensitizing effect of chlorpromazine supported our assumption on the key role of HSP27 in intrinsic W1CR cisplatin resistance.

This highlights HSP27 as the key to the cisplatin resistance of W1CR cells, elucidating the difference between W1 and W1CR cells on a functional level.

### 2.8. Downstream Effects on Caspases and MMPs

**Figure 6:**
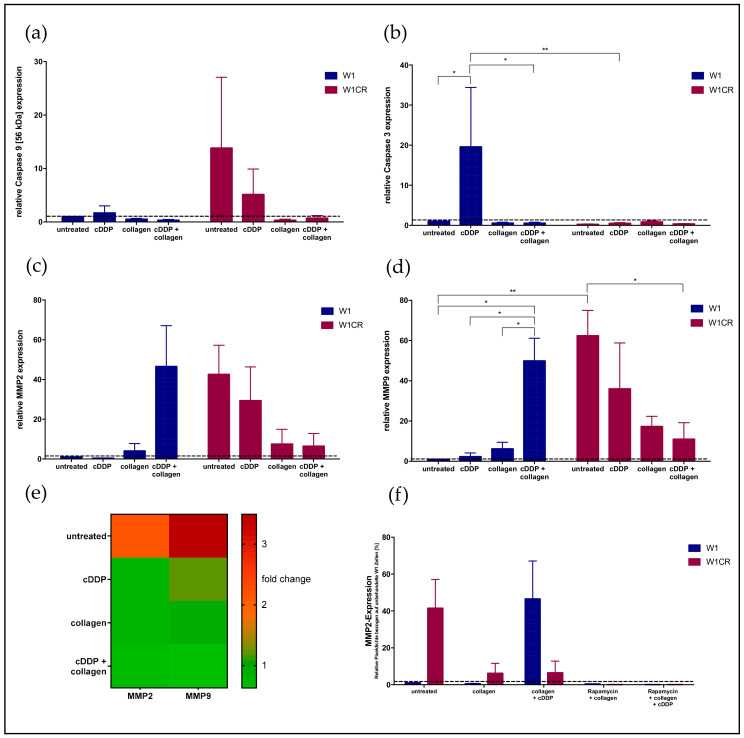
Protein levels of caspase 9 (**a**) and the downstream effector caspase 3 (**b**) in W1 and W1CR cells upon the indicated treatment. Protein levels of MMP2 (**c**) and MMP9 (**d**) in W1 and W1CR cells reflected by the corresponding mRNA levels shown as comparison of W1CR cells versus W1 cells (**e**). (**f**) MMP2 expression levels in W1 and W1CR cells and the impact of a pretreatment with rapamycin thereon. Data are means of at least *n* = 3 (±SEM), asterisks indicate statistical significance: *p < 0.05; **p < 0.01.

To follow the consequences of HSP27 for attenuated cell apoptosis and higher resistance, we focused downstream on caspases. The anti-apoptotic activity of HSP27 is mediated by attenuated caspase activities, especially by blockade of the proteolytic activation of caspase 3 by caspase 9. Our data confirm this attenuated activation axis. Although W1CR exceeded W1 cells with respect to caspase 9 expression, considering the full-length caspase 9 at 56 kDa more than ten-fold (Figure 6a), cleaved caspase 3 (19 kDa) is less detectable in W1CR cells compared to W1 cells (Figure 6b). Notably, W1 cell response toward treatment was clearly indicated at the caspase 3 level, since cisplatin induced a clear caspase 3 upregulation, which is attenuated by COL1. In contrast, W1CR cells resisted cisplatin without the upregulation of caspase 3, indicating the relevance of this pathway, in which COL1 had no further effect.

It is known that HSP27 can enhance cell adhesion and modulate cell migration and invasion via matrix metalloproteases, especially MMP2 and MMP9. In particular, the untreated W1CR cells showed an enhanced expression of MMP2 and MMP9, compared to W1 cells, which can be regarded as a further downstream effect of the HSP27 activity in the W1CR cells (Figure 6c,d). This was also detectable at the mRNA level, where MMP2 and MMP9 were upregulated 2.2- and 3.5-fold, respectively in the untreated W1CR cells compared to W1 cells, indicating the intrinsic nature of this activity (Figure 6e).

Remarkably, W1 cells displayed an upregulated MMP2 and MMP9 level only under COL1 and cisplatin treatment, while similar treatment diminished the MMP2 and MMP9 proteins in W1CR cells. This was also detectable at the mRNA level, where W1CR cells show a much lower expression upon COL1 compared to W1 cells (Figure 6e).

However, the upregulation of MMP2 upon COL1 in W1 cells seemed to be related to the mTOR activity in these treatments (indicated in Figure 1b). To validate the relevance of an mTOR/MMP2 axis, we analyzed the MMP2 expression upon mTOR inhibition using rapamycin in both cell lines. As indicated in Figure 6f, the blockade of mTOR attenuated the MMP2 level of W1 cells further confirming the role of mTOR as a resistance factor in CAM-DR. In contrast, rapamycin had no effect on MMP2 levels in W1CR cells indicating the regulation of MMP2 via another, probably, HSP27 pathway.

## 3. Discussion

In this study we show the complexity of drug resistance in cancer cells that can be mediated by various factors simultaneously. Considering the strong impact of cell-matrix interaction on tumor cell resistance (CAM-DR) we provide here an insight into the signaling pathways of W1 ovarian cancer cells to circumvent cisplatin toxicity. Notably, W1 cells and the constitutively cisplatin resistant cell variant W1CR resist cisplatin toxicity by binding to COL1 obviously in a comparable manner resulting in duplicated EC_50_ values. Here we show that the underlying signaling pathways differ strongly, suggesting a much higher adaptation of W1CR cells to maintain matrix contacts and transfer them into resistance. We were able to decipher CAM-DR and constitutive cisplatin resistance in W1CR cells at the signaling level as two independent, but additive processes based on the MAPK pathway. Within all accepted and known restrictions associated with in vitro cell model studies, our data might be helpful to define known, and accepted targets in cancer treatment for sensitization in a novel context of combined consideration of resistance mechanism.

We show that CAM-DR in W1 cells binding to COL1 is strictly dependent on ITGB1 activity following a p-PRAS/mTOR pathway, confirmed by a knockdown approach of this integrin. PI3K/mTOR is a major growth signaling pathway also involved in drug resistance of ovarian cancer cells. Although mTOR has been reported as important target for sensitization of ovarian cancer cells for chemotherapy, it has not yet been considered in context of CAM-DR. In a recent study, the inhibition of mTORC1/2 by vistusertib (AZD2014) was shown to sensitize various ovarian cancer cell lines for paclitaxel toxicity in a synergistic manner in vitro and in a xenotransplant model of A2780cisR cells [49]. This combination is an issue of an ongoing phase I trial for treatment of patients with ovarian or squamous cell lung cancer (NCT02193633). Other reports associate the dual inhibition of PI3K/mTOR with a strong impact on suppression of cell proliferation, induction of cell cycle arrest and apoptosis in ovarian cancer cells, and the sensitizing for treatments with paclitaxel or cisplatin [50]. Here we confirm that the mTOR inhibitor rapamycin reverses the CAM-DR activity in W1 cells completely and thus, nullifies the effects of COL1 binding on increased EC_50_ values of cisplatin. Since rapamycin has been used at sub-toxic concentrations of 10 nM and 100 nM, these data confirm the value of a co-treatment using mTOR inhibition as a matter to circumvent matrix effects on chemoresistance.

In contrast, rapamycin was not effective in W1CR cells, indicating efficient escape strategies and/or other downstream signals induced by COL1 binding. Since W1CR compensated an ITGB1 knockdown completely maintaining CAM-DR, our findings on the strongly upregulated DDR1 activity in response to COL1 refer to an efficient escape strategy and confirm the high dynamics of cell adaptation in this resistant cell variant. DDR1 has been suggested to serve as potential biomarker for advanced ovarian cancer [51] and has been related to increased cisplatin resistance in those cancer cells [52]. DDR1 induces its cellular effects via different downstream signaling routes, among which the PI3K/AKT and the RAS/MAPK pathway appear to be dominant. This could explain the strong impact of PI3K inhibition reversing the COL1 effect in W1CR cells, both in the scrambled or ITGB1 knockdown variant confirming independence on ITGB1. Referring to MAPK on the other hand, we could show recently that blockade of DDR1 and MAPK components act synergistically in sensitizing breast cancer cells for doxorubicin and mitoxantrone toxicity upon cell binding to collagen [28,53]. Although we could detect intrinsically higher expressions of proteins of the ERK/MEK signaling pathway such as p-MEK and p-CREB in W1CR than in W1 cells, inhibition of these did not lead to an increased sensitivity against cisplatin (Figure S4). A compensatory upregulation of other proteins that ensure cell survival, which has been described for MAPK pathway inhibition before is potentially responsible for the observed effects [54]. We expect this in case of ERK inhibition, which did not lead to a sensitization against cisplatin in the W1CR although displaying higher levels of p-ERK.

Following DDR1-induced MAPK signaling downstream, JNK1/2 were identified as critical components in W1CR cells in response to COL1. Although mRNA upregulation of JNK was not expected, we detected a two-fold upregulation due to COL1 binding which was also reflected on the protein level.

JNK appears intimately involved in coordinating internal cell stress responses to anticancer drugs. Thus, JNK has been considered as an efficient escape route when blocking MAPK components, such as RAF or MEK, indicating an interlink to both pathways. In a recent study, the key role of JNK as mediator of cisplatin and paclitaxel resistance in ovarian cancer has been reported [32]. Of outstanding interest in our context, JNK has been shown to be stimulated by ECM binding of tumor cells, which mediates the drug resistance, e.g., against a RAF inhibitor to breast cancer cells upon binding to collagen [55]. JNK was shown to directly promote breast cancer cell survival by phosphorylating Bcl2 and BclX proteins which protect mitochondrial integrity and counteract apoptosis. In ovarian cancer, the mechanism involves upregulation of survivin, an anti-apoptotic protein that protects stem cell viability [56]. However, our data indicate a serious role of JNK1/2 in mediating the COL1 effect on resistance in W1CR cells, obviously as a downstream key of DDR1 activity. Notably, blocking JNK1/2 by tanzisertib reverses the COL1 effect in these cells but did not affect the constitutive resistance, when comparing to W1 cells. This is a clear indicator for an independent regulation of CAM-DR besides the existing cisplatin resistance in W1CR cells.

HSP27 was identified as a key component in W1CR cells responsible for intrinsic cisplatin resistance, confirmed by a siRNA-mediated knockdown approach. Impressively, HSP27 knockdown turns resistance back to the sensitivity level of the W1 cells. HSP27 has been described in relation to ovarian cancer therapy resistance before, whereby its cytoprotective properties have been associated with modulating oxidative stress and apoptotic signaling [57]. In addition, HSP27 can prevent apoptosis by inactivating Bax. One of the resulting effects of HSP27 activity is an increase in glutathione levels [38,45], which is important for the detoxification of cisplatin. We detected intrinsically higher levels of glutathione in W1CR cells before [9], which might be a reason for the complete sensitization for cisplatin of these cells upon HSP27 knockdown. Small HSPs have received increasing attention in recent years, mainly because of their potential for protective approaches. However, although HSP27 appears a promising target for sensitization strategies, small molecule inhibitors were rare and mainly “side” effects of other drugs, such as the antiviral compounds brivudin or the antipsychotic drug chlorpromazine have been utilized [48] leaving perspectives for further developments. Using the latter drug, we could achieve a certain return in the W1CR cell resistance, not as impressive as using the HSP27 knockdown, which could be due to its non-selectivity. However, our data clearly indicate the key role of HSP27 in intrinsic cisplatin resistance of W1CR cells as an independent way not directly affected, but acting additionally to the CAM-DR phenomenon. Furthermore, the small chaperon interacts with the outer mitochondrial membranes and interferes with the activation of the cytochrome c/Apaf-1/dATP complex, thereby inhibiting the activation of procaspase-9. The impact of increased activity of HSP27 on attenuated activation of caspase cascade appears as a final answer for increased survival of W1CR cells under cisplatin stress.

Interestingly, previous studies also showed that the collagen/DDR1 axis can induce the expression and activation of MMPs [26]. Recently, first studies confirmed a common interaction site of HSP27 in glioblastoma for both MMP2 and MMP9 [58]. Here we were able to detect this link for the first time in ovarian cancer cells. Concerning W1 wild-type cells, we observed an increase in MMP levels upon COL1 and cisplatin treatment. This confirms a mTOR/MMP axis reflecting the increased activation of the mTOR pathway in these cells, which has also been shown for several other cancers [59,60]. Furthermore, the downregulation of the intrinsically higher levels of MMP2 and MMP9 by COL1 in resistant W1CR cells confirm the discrete regulation of both resistance pathways and offer interesting new targets for clinical sensitization strategies in the treatment of cancer patients.

## 4. Materials and Methods

### 4.1. Cell Culture

The human ovarian carcinoma cell line W1 and the cisplatin-resistant W1CR subtype, which was achieved by exposing W1 cells to cisplatin at incrementally elevated concentrations were cultivated at 37 °C and 5% CO_2_ in RPMI 1640 medium containing 10% FCS and 1% penicillin, as described before [6]. Cells were detached using a solution of EDTA (0.2 g/L EDTA × 4 Na) for 2 min at 37 °C. To ensure the resistance for a longer period of time, 1 mg/mL cisplatin was added to the W1CR cell medium. All cell culture reagents were from PAN Biotech GmbH, Aidenbach, Germany. The preservation of cisplatin resistance in W1CR cells, as well as the absence of mycoplasma in cell culture was verified every second week.

The 96-well plates were coated with COL1 (Corning, Thermo Fisher Scientific Inc., Waltham, MA, USA) at a density of 10 μg/cm^2^, according to the manufacturer’s protocol. For Western blot experiments, collagen-coated cell flasks were used (Sarstedt AG & Co, Nümbrecht, Germany). BEZ235 (dactolisib), a dual pan-PI3K/mTOR small molecule inhibitor was obtained from Selleck Chemicals (Houston, TX, USA). Cells were treated with 10 nM BEZ235, for 6 h, before the start of the MTT assay. LY294002, a selective PI3K inhibitor (Abcam, Cambridge, UK) was applied at a concentration of 0.1 µM with the same incubation time as BEZ235. For the inhibition of FAK phosphorylation at Y397, a FAK inhibitor 14 (Biomol, Hamburg, Germany) was added to the cells at a concentration of 1 µM, 4 h before conducting MTT experiments. Rapamycin (Santa Cruz Biotechnology, Heidelberg, Germany) was used in the experiments as a mTOR inhibitor in concentrations of 10 nM and 100 nM. Inhibitors of the MAPK signaling pathway were investigated including MEK inhibitor U0126 (Selleck Chemicals) at a concentration of 0.5 µM, ERK1/2 inhibitor SCH772984 (Hycultec, Beutelsbach, Germany) at 0.01 µM, CREB inhibitor 666-15 (Hycultec) at 0.1 µM, JNK inhibitor tanzisertib (MedChemExpress, Monmouth Junction, NJ, USA) at 1 µM and non-selective HSP27 inhibitor chlorpromazine (Merck, Darmstadt, Germany) at 10 µM. The indicated inhibitors were added to the cells 4 h prior to cytostatic treatment.

### 4.2. β1-Integrin Knockdown of W1 and W1CR Cells

ITGB1 of W1 and W1CR cells was knocked down using viral transduction as described before [9]. Cells were seeded in a 96-well plate at a density of 1000 cells per well and incubated overnight in RPMI. The next day, the medium was substituted with 100 µL per well of transduction medium (Polybrene^®^ 4μg/mL in complete cell medium). Also the viral particles (control shRNA lentiviral particles-A and ITGB1 shRNA (h) lentiviral particle: sc-35674-V; Santa Cruz Biotechnology, Heidelberg, Germany) were thawed and resuspended. In one well, 4 μL of ITGB1 shRNA (h) lentiviral particles were added to the medium, the same procedure was carried out for the control shRNA lentiviral particles in another well. After one day, media were changed and medium containing the still infectious viral particles were used to transfect another well in the same manner. For selection of stable clones, cells were incubated in medium containing 2.75 µg/mL puromycin (Carl Roth GmbH, Karlsruhe, Germany). Knockdown was confirmed by Western blot using a mouse anti-β1-integrin antibody [P2D5] (Santa Cruz Biotechnology).

### 4.3. Small-Interfering (siRNA) RNA Transfection of W1CR Cells to Knockdown HSP27

siRNA transfection was performed with the siRNA reagent system (sc-45064, Santa Cruz Biotechnology) following the manufacturer‘s instructions. Briefly, 2 × 10^5^ cells/well were replanted in six-well plates in antibiotic-free medium overnight or until they reached 80% confluence. For each transfection, 6 µL of HSP27 siRNA or unconjugated control siRNA-A in 100 µL of transfection medium was mixed with 6 µL of transfection reagent (all materials from Santa Cruz Biotechnology) in another 100 µL of transfection medium. The mixtures were incubated for 30 min at room temperature. Each well was washed with 2 mL transfection medium. After incubation overnight, 1 mL of medium supplemented with 20% FBS with 2% antibiotics was added to each well for another 24 h. The transfection mixture was removed and replaced with normal growth medium on the following day. All experimental measurements were performed 24 h after medium replacement.

### 4.4. Cytotoxicity Assay

MTT assays were used to measure the cytotoxicity of cisplatin (Sigma-Aldrich Chemie GmbH, Steinheim, Germany) in W1 and W1CR cells and respective effects of the indicated inhibitors using 3-(4,5-dimethylthiazol-2-yl)-2,5-diphenyltetrazolium bromide (BioChemica, Applichem GmbH, Darmstadt, Germany) as described in [61]. Cells were seeded in triplicates in normal 96-well plates at a density of 5 × 10^3^ cells/well and 1 × 10^4^ (Sarstedt AG & Co). Partly, plates were coated with COL1 as described before and preincubated overnight. The following day, cells were treated with an inhibitor, if necessary, and after an appropriate incubation period supplemented with a dilution series of cisplatin (10^−3.5^ to 10^−7.5^ M). 72 h after cisplatin addition the MTT solution (20 µL, 5 mg/mL) was added into the wells for 1 h at 37 °C and 5% CO_2_ until formazan crystals were formed. The supernatant was removed, and the cells were dissolved in 200 µL DMSO. Absorption was analyzed at 570 nm, with background subtraction at 690 nm, using a plate reader (Thermomultiscan EX, Thermo, Schwerte, Germany).

### 4.5. Western Blot

Cell protein lysates were prepared using the cell extraction buffer (Life Technologies, Carlsbad, CA, USA) as mentioned before [9]. A BCA protein assay kit was used for protein quantification. SDS-Page and Western blots were performed as described using stain-free gels [62]. Membranes were incubated with mouse anti-GAPDH (GeneTex, Irvine, CA, USA), rabbit anti-FAK, rat anti-p-FAK (Y397, R&D Systems GmbH, Wiesbaden-Nordenstadt, Germany) rabbit anti-PI3K, goat anti-p-PI3K (Tyr 508), goat anti-AKT1, rabbit anti-p-AKT1 (Tyr308), mouse anti-PTEN, mouse anti-p-PTEN (Ser380), mouse anti-mTOR, mouse anti-p-mTOR (Ser2448), mouse anti-HSP27, mouse anti-p-HSP27 (Ser82), rabbit anti-DDR, rabbit anti-p-DDR (Tyr513, Cell Signaling Technology, Frankfurt am Main, Germany), mouse anti-CREB, mouse anti-p-CREB (Ser133), mouse anti-JNK, mouse anti-p-JNK (Thr 183 and Tyr185), mouse anti-p-MEK1/2, rabbit anti-ERK1/2, rabbit anti-p-ERK1/2 (Thr202/Tyr204, Cell Signaling Technology), as well as goat anti-rabbit, donkey anti-goat and anti-mouse IgG kappa binding protein IgG HRP-conjugated diluted in 1% BSA solution. If not indicated otherwise antibodies were purchased from Santa Cruz Biotechnology.

Western blots were quantified via chemiluminescence using a Clarity Western ECL substrate chemiluminescence kit (BioRad Laboratories GmbH, Munich, Germany). Besides GAPDH as a loading control, a stainfree total protein normalization was used. Membranes were photographed and analyzed using a ChemiDoc XRS+ imaging acquiring system (BioRad) and Image Lab software v. 6.0 (BioRad).

### 4.6. Proteome ProfilerTM Array

A Proteome Profiler™ Human Phospho-Kinase Array Kit (R&D Systems) was performed to examine W1 and W1CR cells for changes in intracellular signaling pathways comparing untreated cells with the effects of COL1 binding and cytotoxic stress by cisplatin (EC_50_). Cell lysates from W1 and W1CR cells were prepared and Pierce™ BCA Protein Assay Kit (LifeTechnologies, Thermo Fisher Scientific Inc.) was used to quantify total protein. The assay was performed according to the manufacturer’s instructions. Membranes were photographed and quantified using ChemiDoc XRS+ imaging acquiring system (BioRad), and Image Lab software v. 6.0 (BioRad).

### 4.7. Microarray

To conduct microarray experiments, 100 ng of total RNA of each condition was used as input. Samples were reversely transcribed into cDNA and then in vitro transcribed according to the manufacturer’s protocol using GeneChip™ 3′ IVT PLUS Reagent Kit (Applied Biosystems, Thermo Fisher Scientific Inc.). Next, samples were fragmented and hybridized on Affymetrix GeneChip human genome U219 microarrays, together with control cRNA and oligo B2. Hybridization was conducted at 45 °C for 16 h, using an AccuBlock™ Digital dry bath (Labnet International, Inc., New York, NY, USA) hybridization oven. Further, the microarrays were washed and stained according to the manufacturer’s protocol using an Affymetrix GeneAtlas™ Fluidics Station (Affymetrix, Santa Clara, CA, USA). In the final step, all microarrays were scanned using an Affymetrix GeneAtlas™ imaging station (Affymetrix). The scans of the microarrays were saved as *.CEL files on local storage. Downstream analysis was conducted in R/RStudio IDE (R – version 4.0.2, RStudio – version 1.3.1056), Bioconductor (version 1.3.10) as well as Transcriptome Analysis Software 4.0.1.36. Microarray results were stored in the GEO database under ID GSE140996.

### 4.8. Statistics

MTT results of the sigmoidal dose-response curves were evaluated by a nonlinear regression using the four-parameter logistic equation with variable hill slope to determine the EC_50_ at the curves’ inflection point and to acquire sigmoidal dose-response curves (GraphPad 6.0 Software, San Diego, CA, USA). Moreover, statistical analysis was performed using one-way ANOVA following Tukey’s multiple comparison test (* *p* < 0.05; ** *p* < 0.01; *** *p* < 0.001; **** *p* < 0.0001).

## 5. Conclusions

Our study provides an insight into the relevance and complexity of CAM-DR in W1 ovarian cancer cells offering novel targets for this independently regulated, and not yet well-addressed impact of matrix on cancer susceptibility in the clinical treatment. In these turns, mTOR and JNK1/2 have been identified as promising targets in W1, and W1CR cells, respectively, to overcome CAM-DR. Moreover, we were able to highlight HSP27 as key for intrinsic cisplatin resistance in W1CR cells emerging this chaperone as an attractive target for sensitization strategies and recommend this molecule as a pattern for a challenging design and development of potential inhibitors. Concerning the model character of these investigations including restrictions for further generalization, continuing studies are needed using other tumor cells to verify these findings on CAM-DR before preclinical in vivo studies have to confirm the value of these sensitization targets and strategies.

## Supplementary Materials

The following supplementary figures are available online at https://www.mdpi.com/1422-0067/21/23/9240/s1, Figure S1: Knockdown of ITGB1 in W1 and W1CR cells. Figure S2: The impact of PI3K and PTEN pathway on the resistance of W1 and W1CR cells. Figure S3: Western blot data of the wnt signaling related p-GSK3α/β. Figure S4: The influence on MAPK pathway in W1 and W1CR cells. Figure S5: The role of CREB, a MAPK pathway related downstream component in W1 and W1CR cells to regulate the sensitivity to cisplatin.

## Abbreviations

AKT	protein kinase B
CAM-DR	cell adhesion mediated drug resistance
cDDP	cisplatin
COL1	collagen type 1
DDR1/2	discoidin domain receptor 1/2
ERK	extracellular signal regulated kinase
FAK	focal adhesion kinase
HSPs	heat shock proteins
ILK	integrin-linked kinase
ITGB1	integrin β1
JNK	c-Jun-N-terminal kinase
kd	knockdown
MMP	matrix metalloprotease
mTOR	mechanistic target of rapamycin
PI3K	phosphoinositide-3-kinase
RTK	receptor tyrosine kinases
