# Supplementary material for: Insight into Cisplatin-Resistance Signaling of W1 Ovarian Cancer Cells Emerges mTOR and HSP27 as Targets for Sensitization Strategies
Source: Int J Mol Sci. 2020 Dec 3;21(23):9240. doi: 10.3390/ijms21239240 (PMC7730285; doi:10.3390/ijms21239240)
Supplement: Supplementary file 1 [file ijms-21-09240-s001.pdf]

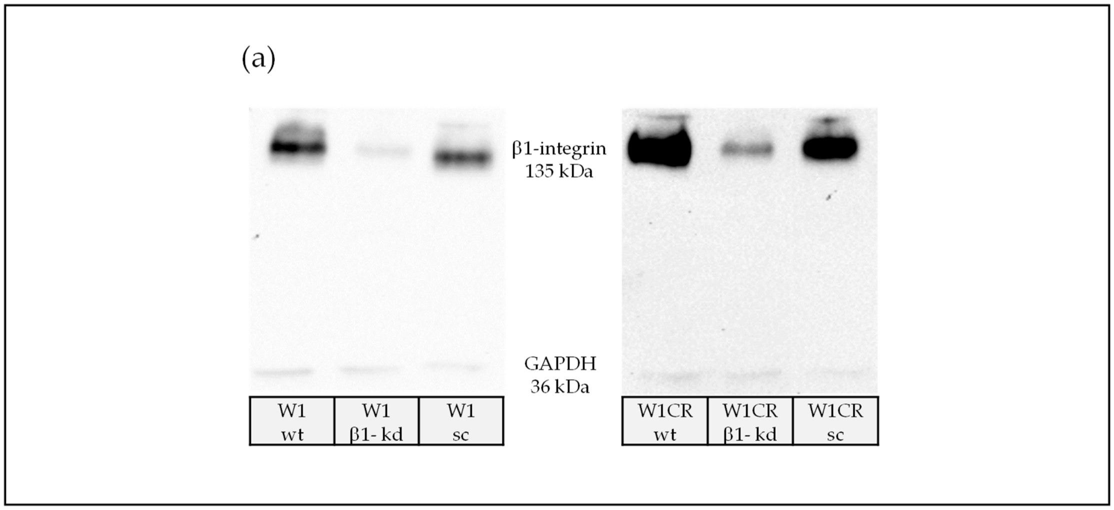

Figure S1: Knockdown of ITGB1 in W1 and W1CR cells. Shown is a representative Western blot of ITGB1 knockdown in W1 and W1CR cells, confirming the almost complete deletion of ITGB1 in the knockdown cells compared to scrambled control.

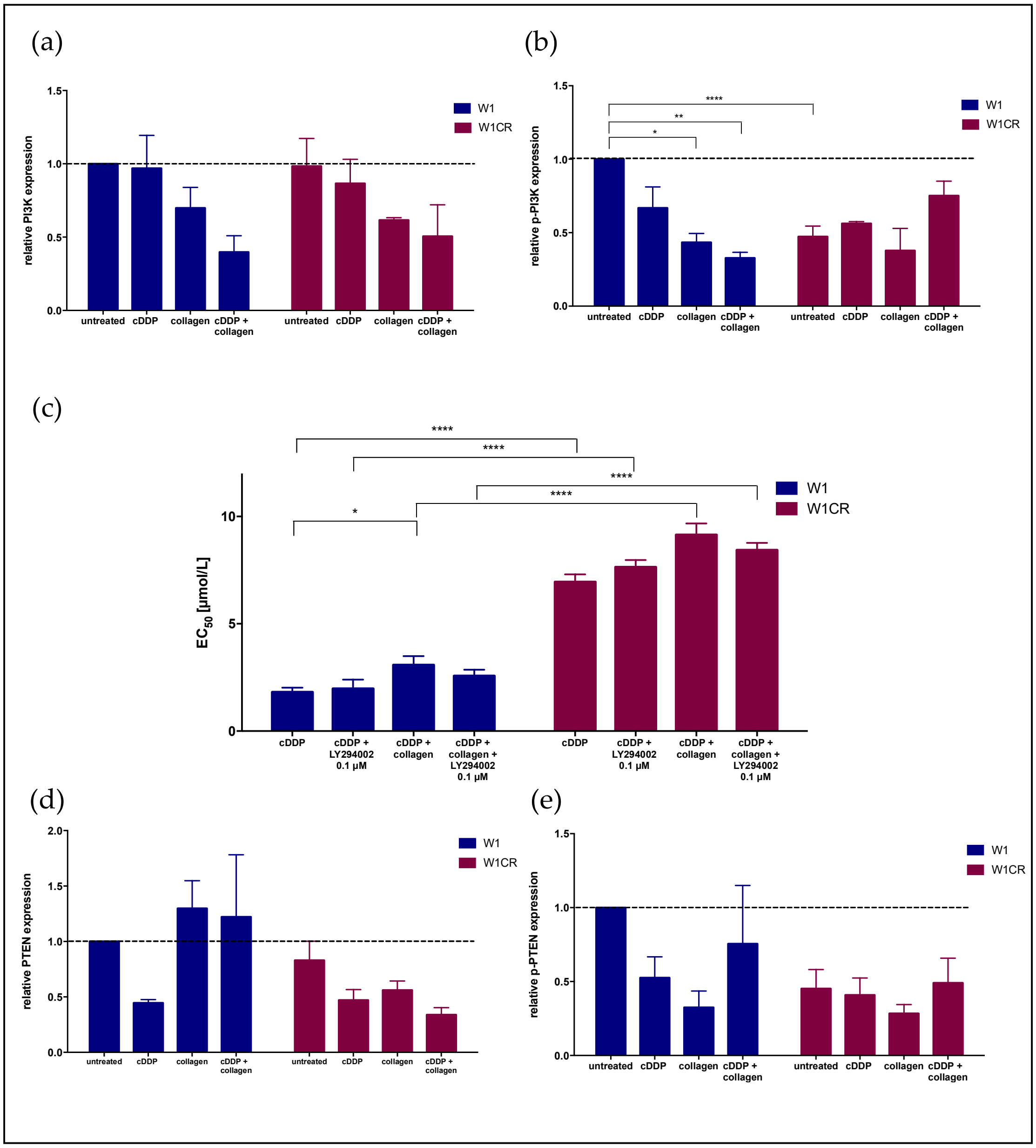

Figure S2: The impact of PI3K and PTEN pathway on the resistance of W1 and W1CR cells. Western blot data of PI3K (**a**) and phosphorylated fraction of PI3K (**b**). EC_{50} levels under PI3K inhibition with LY294002 (0.1 μM) (**c**). Protein levels of PTEN (**d**) and p-PTEN (**e**). Data are means of at least n=3 (±SEM), asterisks indicate statistical significance: $^{*}p<0.05$; $^{**}p<0.01$; $^{***}p<0.001$; $^{****}p<0.0001$.

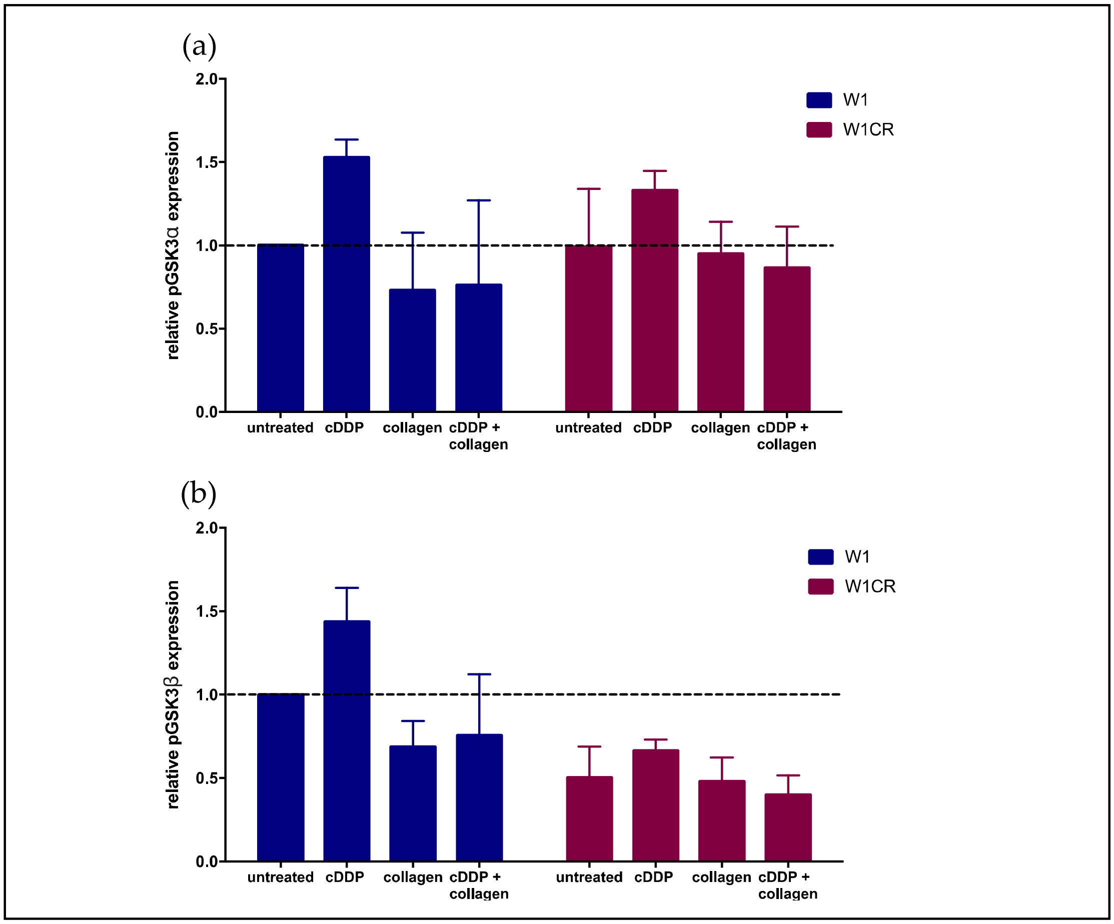

Figure S3: Western blot data of the wnt signaling related p-GSK3α (**a**) and p-GSK3β (**b**) proteins in W1 and W1CR cells and their deregulation by cisplatin and/or collagen treatment.

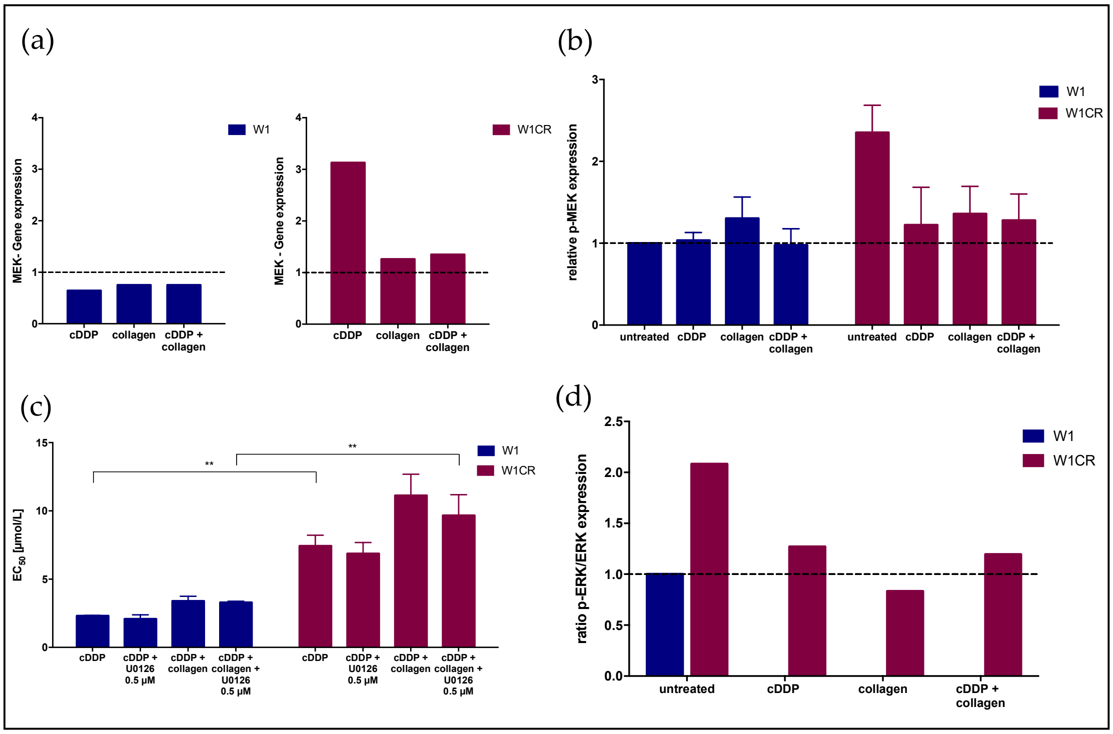

Figure S4: The influence on MAPK pathway in W1 and W1CR cells. Deregulation of gene expression (fold change) of MEK **(a)** in W1 and W1CR cells upon the indicated treatment. Expression of p-MEK (**b**) at protein level and inhibition of p-MEK by U0126 at 0.5 µM (**c**). (**d**) Protein expression of ERK given as the p-ERK/ERK ratio as an indicator of ERK signaling activity. Protein data are means of at least n=3 (±SEM), asterisks indicate statistical significance: $^{*}p<0.05$; $^{**}p<0.01$.

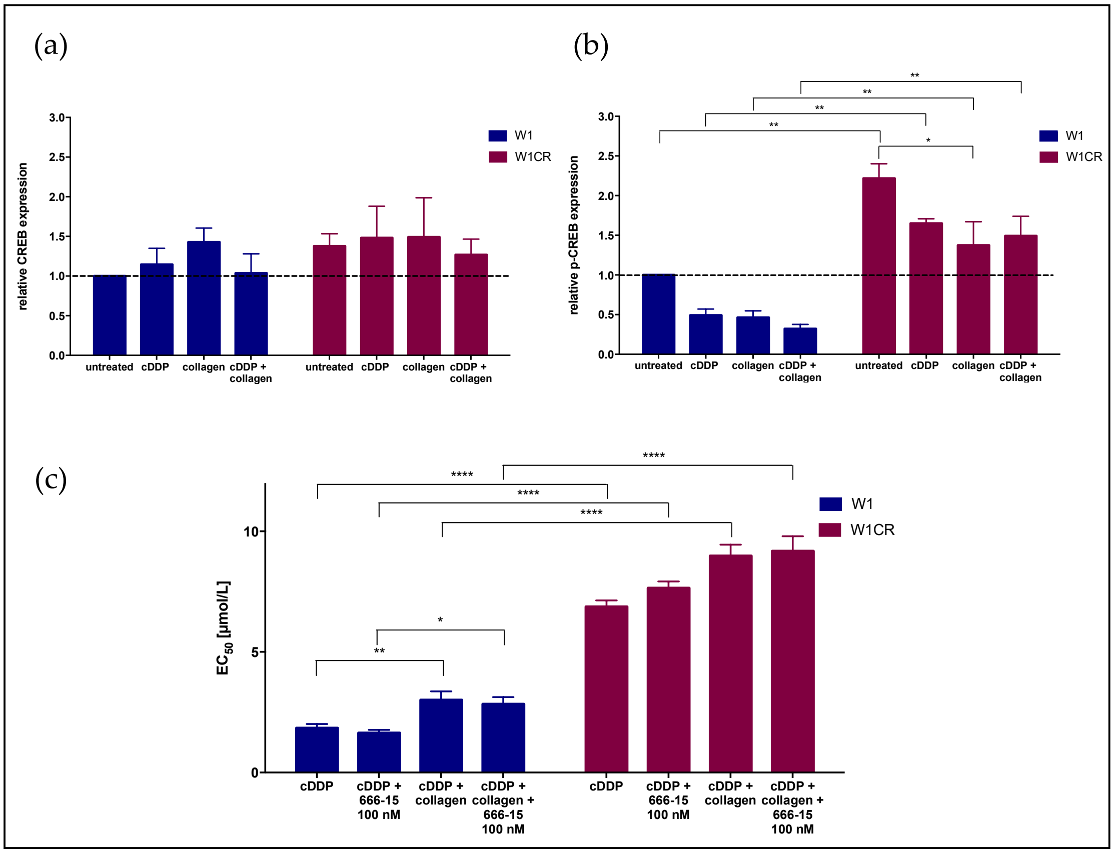

Figure S5: The role of CREB, a MAPK pathway related downstream component in W1 and W1CR cells to regulate the sensitivity to cisplatin. Western blot data of CREB (**a**) and p-CREB (**b**) in W1 and W1CR cells upon the indicated treatments. (**c**) Impact of the CREB inhibitor 666-15 (0.1 µM) to affect the cisplatin sensitivity in both cell lines. Data are means of at least n=3 (±SEM), asterisks indicate statistical significance: $^{*}p<0.05$; $^{**}p<0.01$; $^{***}p<0.001$; $^{****}p<0.0001$.
